# The anterior temporal lobes support residual comprehension in Wernicke’s aphasia

**DOI:** 10.1093/brain/awt373

**Published:** 2014-02-10

**Authors:** Holly Robson, Roland Zahn, James L. Keidel, Richard J. Binney, Karen Sage, Matthew A. Lambon Ralph

**Affiliations:** 1 Neuroscience and Aphasia Research Unit, School Psychological Sciences, University of Manchester, UK; 2 School of Psychology and Clinical Language Sciences, University of Reading, UK; 3 Department of Psychological Medicine, Institute of Psychiatry, King's College London, UK; 4 School of Psychology, Bangor University, UK; 5 Faculty of Health and Social Care, University of the West of England, Bristol, UK

**Keywords:** Wernicke’s aphasia, semantic processing, language comprehension, anterior temporal lobe, Wernicke’s area

## Abstract

Wernicke’s aphasia occurs after a stroke to classical language comprehension regions in the left temporoparietal cortex. Consequently, auditory–verbal comprehension is significantly impaired in Wernicke’s aphasia but the capacity to comprehend visually presented materials (written words and pictures) is partially spared. This study used functional magnetic resonance imaging to investigate the neural basis of written word and picture semantic processing in Wernicke’s aphasia, with the wider aim of examining how the semantic system is altered after damage to the classical comprehension regions. Twelve participants with chronic Wernicke’s aphasia and 12 control participants performed semantic animate–inanimate judgements and a visual height judgement baseline task. Whole brain and region of interest analysis in Wernicke’s aphasia and control participants found that semantic judgements were underpinned by activation in the ventral and anterior temporal lobes bilaterally. The Wernicke’s aphasia group displayed an ‘over-activation’ in comparison with control participants, indicating that anterior temporal lobe regions become increasingly influential following reduction in posterior semantic resources. Semantic processing of written words in Wernicke’s aphasia was additionally supported by recruitment of the right anterior superior temporal lobe, a region previously associated with recovery from auditory-verbal comprehension impairments. Overall, the results provide support for models in which the anterior temporal lobes are crucial for multimodal semantic processing and that these regions may be accessed without support from classic posterior comprehension regions.

## Abstract (precis)

Robson *et al*. use fMRI to investigate preserved written word and picture comprehension in Wernicke’s aphasia (impaired verbal comprehension following left temporoparietal damage). Bilaterally enhanced activation in the ventral and anterior temporal lobes as patients semantically process visually presented material emphasizes the importance of these regions for multimodal comprehension.

## Introduction

Wernicke’s aphasia is the classical aphasic syndrome associated with impaired language comprehension. Wernicke’s aphasia results from lesions to the left posterior temporoparietal cortex (Bogen and Bogen, 1976; Dronkers *et al.*, 1995), thereby affecting core elements of the phonological and semantic systems (Robson *et al.*, 2012*a*, 2013) that interact during language comprehension. The comprehension impairment in Wernicke’s aphasia is modulated by the degree of phonological analysis required (Robson *et al.*, 2012*b*). Spoken language comprehension, which requires a high degree of phonological analysis for word recognition, is severely impaired in Wernicke’s aphasia. Written word comprehension, which is mediated by both phonological and visual processes, is significantly less impaired in Wernicke’s aphasia in comparison with spoken word comprehension. Comprehension of pictorial materials, which primarily requires visual analysis processes, is comparatively preserved (although not necessarily intact) in Wernicke’s aphasia (Robson *et al.*, 2012*b*). Given that semantic cognition is distributed over a number of perisylvian and extra-sylvian regions (Jefferies and Lambon Ralph, 2006), it is of clinical and neurobiological importance to determine the functional anatomy that supports residual semantic processing of visually-presented items (pictures and written words) in a group of homogenous, classical Wernicke’s aphasia participants.

Recent functional imaging studies involving participants with variable levels of recovered aphasia have demonstrated the importance of anterior temporal lobe regions for language comprehension, in particular the anterior fusiform gyrus, superior anterior temporal lobe and temporal pole. Sharp *et al.* (2004) used an auditory semantic association task in a functional PET investigation to assess individuals with lesions to the left superior temporal lobe who had recovered single word comprehension. Increased activation was found in the right anterior fusiform gyrus and bilateral temporal poles. Functional MRI studies using passive narrative listening have also emphasized the role of the superior anterior temporal lobes. Activation in the right superior anterior temporal lobe during narrative listening was found to correlate with measures of sentence comprehension in individuals with a history of aphasia (Crinion and Price, 2005) and the degree of interhemispheric functional connectivity between the superior anterior temporal lobes was found to correlate with single word and sentence comprehension measures in participants with chronic aphasia (Warren *et al.*, 2009).

The evidence that the anterior temporal lobes support comprehension in aphasia recovery is consistent with other lines of research that highlight the anterior temporal lobes as crucial components of the semantic network; specifically, regions that support abstraction of transmodal representational semantic knowledge (Patterson *et al.*, 2007; Lambon Ralph *et al.*, 2010). The strongest evidence for anterior temporal lobe involvement in semantic representation has emerged from neuropsychological investigations of semantic dementia patients, who have progressive atrophy focussed on the anterior temporal lobes bilaterally and a resultant progressive degradation of semantic representations (Mummery *et al.*, 2000; Patterson *et al.*, 2007). Additionally, repetitive transcranial magnetic stimulation to the right or left anterior temporal lobe in neurologically-normal young controls slows reaction times for semantic but not numerical judgements (Lambon Ralph *et al.*, 2009; Pobric *et al.*, 2010*a*, *b*) and neuroimaging studies (which avoid or correct for signal dropout) have shown responses in the anterior temporal lobes during multimodal semantic processing tasks (Vandenberghe *et al.*, 1996; Marinkovic *et al.*, 2003; Liu *et al.*, 2009; Binney *et al.*, 2010; Visser *et al.*, 2010*a*).

These lines of research suggest that anterior temporal regions support core conceptual knowledge and that these areas may become increasingly influential when posterior areas of the language network are damaged. Such notions contrast with traditional neurobiological models of language (Geschwind, 1965; Geschwin, 1972). Based on deficits associated with Wernicke’s aphasia, traditional models have emphasized the role of the posterior temporoparietal region as the central access point to, or locus for the representation of semantic knowledge. Current neurobiological models of language implicate a wider, distributed lateral and medial temporal–parietal–frontal network, within which the temporoparietal region remains a core element (Binder *et al.*, 2009).

Although we have emphasized here the importance of anterior temporal lobe regions in semantic representation, it should be noted that the ‘hub-and-spoke’ model of semantic representation (Rogers *et al.*, 2004; Patterson *et al.*, 2007; Lambon Ralph *et al.*, 2010) builds on previous notions that concepts derive from anterior temporal lobe coordinated activation of information encoded in a set of distributed modality-specific association areas (the ‘spokes’). Meynert and Wernicke’s view of conceptualization suggested that only the distributed modality-specific regions were necessary. Direct descendants of these ideas are found in the modern literature and captured in the hypothesis of ‘embodied cognition’ (Barsalou *et al.*, 2003), which can vary in form from weak to strong formulations (Meteyard *et al.*, 2012). Again, the key idea in these theories is that concepts reflect the mass action of multiple information sources that are experienced and encoded in each modality, separately. Considerable evidence for this approach has come from functional neuroimaging and neuropsychological studies (Pulvermuller, 2005; Martin, 2007; Kemmerer *et al.*, 2012). The hub-and-spoke hypothesis suggests that coherent concepts require both transmodal anterior temporal lobe representations plus these distributed modality-specific sources of information (for discussion of these issues, see: Patterson *et al.*, 2007; Lambon Ralph, 2013). The combined roles of transmodal anterior temporal lobe and modality-specific regions in semantic processing have been confirmed by utilizing transcranial magnetic stimulation to investigate and compare different neural regions within the same neurologically-intact participants (Pobric *et al.*, 2010*a*).

In contrast with the majority of neuroimaging studies of aphasia, this study focused on a group of individuals with classical chronic Wernicke’s aphasia. We used distortion-corrected functional MRI to investigate semantic processing with the aims of revealing important insights into the clinical manifestation of Wernicke’s aphasia and the neural basis of semantic cognition. Specifically, we explored: (i) the neural regions underlying semantic processing in Wernicke’s aphasia; and (ii) how the semantic system adapts to the removal of core posterior components. Participants with chronic Wernicke’s aphasia and age-matched controls made semantic judgements about single items which engage minimal or moderate phonological processing (pictures and written words, respectively, in comparison with spoken word processing). Based on previous findings in aphasia and the theory that the anterior temporal lobes support representational semantics, it was hypothesized that participants with Wernicke’s aphasia should demonstrate significant activation of the anterior temporal lobe when semantically processing visually-presented materials (picture or written words). Because lesions in Wernicke’s aphasia affect phonological processing regions of the left superior temporal lobe, it was hypothesized that written words processing would show a greater degree of reorganization in comparison with picture processing and engage superior temporal regions of the right hemisphere. We tested these hypotheses in 12 individuals with classical Wernicke’s aphasia and 12 control participants. To ensure complete and reliable coverage of all anterior temporal regions, we used distortion-corrected functional MRI which minimizes signal loss and distortion over these areas (Embleton *et al.*, 2010).

## Materials and methods

### Participant diagnosis and lesions

Twelve participants with chronic Wernicke’s aphasia [two female, mean age 70.1, standard deviation (SD) 8.7] and 12 age- and education-matched control participants (one female, mean age 71, SD 6.9) were recruited and provided written informed consent as approved by the Multicentre Research Ethics Committee NHS ethics committee. All participants were right-handed in that they wrote with their right hand. Control participants were screened to ensure they had no previous or current neurological, language or cognitive deficit and all were native speakers of English.

All patients presented with classical symptoms of Wernicke’s aphasia after a single left hemisphere stroke; namely impaired single word comprehension, single word repetition and fluent, sentence-like speech punctuated with phonological or neologistic errors. Diagnosis was confirmed using the Boston Diagnostic Aphasia Examination (Goodglass *et al.*, 2001). Table 1 displays biographical and behavioural diagnostic data. Additional background language data were collected for: single-word spoken versus written comprehension; written word versus picture semantic association judgements; and single word reading aloud. The results are displayed in Table 2. Single word comprehension was assessed using the word-to-picture matching test from the 64-item Cambridge Semantic Battery (Bozeat *et al.*, 2000). In this test a spoken or written word is presented and the participant is asked to select the matching item from a set of 10 semantically-related pictures. The spoken and written versions use the same items and differ only on modality of presentation. Semantic association judgements were assessed using the Pyramids and Palm Trees test (Howard and Patterson, 1992). In this test the participant must judge which of two semantically related items is associated with a probe item. Two versions of the test were administered, in which the same triads are presented either as written words or pictures. Single-word reading aloud was taken from the Psycholinguistic Assessment of Language Processing in Aphasia (PALPA: Kay *et al.*, 1992). Consistent with previous reports (Robson *et al.*, 2012*a*), test accuracy varied with the degree of phonological processing required, so that semantic association judgements were significantly more accurate for pictures than written words [*t*(11) = 2.8, *P = *0.017] and single word comprehension was significantly more accurate for written than spoken words [*t*(10) = 5.4, *P < *0.001]. Table 2 summarizes the results from these assessments for each participant. All participants displayed impaired spoken single word comprehension and 7/11 participants displayed impaired written word comprehension. Seven participants were impaired at picture semantic association and eight participants were impaired at written word semantic association. The participants who were unimpaired at semantic association displayed the least severe comprehension impairment overall.

**Table 1:** Demographic and diagnostic assessment for Wernicke’s aphasia and control participants

WA	Age	Sex	Time post-onset (months)	Aetiology	Lesion	BDAE comprehension		BDAE fluency	BDAE repetition		Control	Age	Sex	ACE-R	
						Total %	Total max 32	%	Sentence %	Word %				MMSE Max 30	Total max 100
DR	76	M	7	In	pSTL, TPJ, IFL	2	6	47	<1	<1	BR	76	M	26	78*
DMC	67	M	10	Haem	STG, TPJ, MTL, IFL, IPL	3	4	47	<1	<1	BH	67	M	30	98
DL	73	M	9	In	STL, MTL, ATL, IFL	3	5	63	<1	<1	DC	74	M	27	85
LS	66	M	10	In	pSTL, pMTL,IPL	5	10.5	70	25	25	DW	72	M	30	92
LB	80	F	84	In	STL, MTL, TPJ, IPL	5	9	68	5	15	EC	78	F	28	87
CB	59	M	14	In	STL, IPL, IFL, ATL	10	8	38	15	10	TT	61	M	26	91
RD	87	M	17	In	pSTL, IPL, TPJ	10	14	80	5	10	NJ	78	M	30	88
MC	73	F	13	In	pSTL, TPJ, IPL	10	14	83	10	10	HE	76	M	30	98
EL	61	M	15	In	pSTL,MTL, TPJ	14	17	75	10	15	ML	66	M	28	90
NM	59	M	11	In	STL, MTL, TPJ, ATL	17	15.5	100	10	10	AM	58	M	30	96
CH	77	M	17	In	STL, MTL, TPJ	40	25	90	45	5	GP	78	M	26	93
CW	70	M	36	In	STL, MTL, TPJ, IPL	40	26	100	40	15	KW	69	M	30	99

Wernicke’s aphasia (WA) participants were screened and diagnosed using the BDAE (Boston Diagnostic Aphasia Examination). Control participants were screened using ACE-R = Addenbrooks Cognitive Examination – Revised, from which a Mini-Mental State Examination (MMSE) score (Folstein *et al.*, 1975) can be derived. Wernicke’s aphasia participants are ordered by severity of auditory comprehension disorder, most (DR) to least (CW). Aetiology: In = infarct; Haem = haemorrhage. Lesion indicates core regions of left hemisphere cortical and subcortical damage in the Wernicke’s aphasia participants: p* = *posterior; STL = superior temporal lobe; TPJ = temporoparietal junction; IFL = inferior frontal lobe; MTL = middle temporal lobe; ATL = anterior temporal lobe; IPL = inferior parietal lobe.

*Control participants with low level literacy score.

**Table 2:** Background language testing in Wernicke’s aphasia group

		Picture Pyramids and Palm Trees	Written Pyramids and Palm Trees	Written word-to-picture match	Spoken word-to-picture match	PALPA single word reading
PT	*Max*	*52*	*52*	*64*	*64*	80
	*Cut-off*	49	49	63	63	73
DR		*47*	*33*	*33*	*9*	*0*
DMC		*42*	*39*	*28*	*16*	*1*
DL		*46*	*32*	*54*	*8*	*14*
LS		*46*	*34*	N/A	*32*	*0*
LB		*48*	*42*	*58*	*26*	*14*
CB		*42*	*43*	*50*	*30*	*0*
RD		50	52	64	*47*	*4*
MC		50	*47*	*60*	*55*	*21*
EL		*48*	*36*	*59*	*30*	*27*
NM		52	52	*62*	*53*	*28*
CH		51	50	63	*53*	*19*
CW		51	52	64	*51*	*44*
Mean		47.8	42.7	54.1	34.2	14.3
SD		3.3	7.8	12.5	17.4	14.1

Table displays background behavioural semantic and comprehension assessments. *Italics* indicate outside normal limits. PT = participant. The Pyramids and Palm Trees test (Howard and Patterson, 1992) assesses semantic association, word-picture-matching (Bozeat *et al.,* 2000) assesses single word comprehension. Reading score from subtest 31 of PALPA (Kay *et al.*, 1992). N/A = not available.

**Figure 1:**
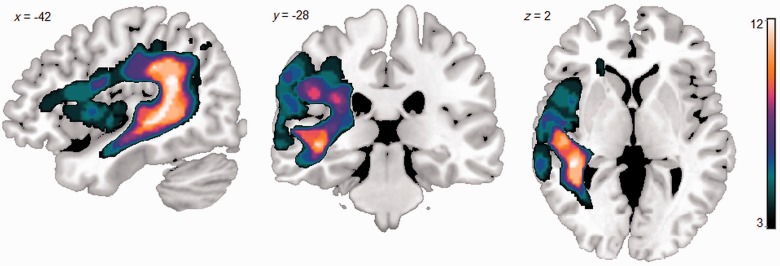
Lesion overlap map for the 12 participants with Wernicke’s aphasia. The lesion distribution mirrors previous studies of Wernicke’s aphasia, with lesions centred on posterior perisylvian cortical and subcortical regions. Colour bar indicates the number of participants with a lesion at each voxel (min = 3; max = 12).

Structural T_1_-weighted magnetic resonance images were acquired before functional MRI scanning on a 3 T Philips Achieva scanner with an eight-element SENSE head coil and a sense factor of 2.5. An inversion recovery sequence produced a 256 × 256 matrix of 128 transverse slices with 1 mm^3^ voxels. Lesions were extracted using the automated lesion identification algorithm (Seghier *et al.*, 2008) and overlaid to produce a lesion overlap map (Fig. 1). Maximal lesion overlap occurred in the white matter underlying the posterior superior temporal lobe, consistent with the classical description of Wernicke’s aphasia (Bogen and Bogen, 1976). However, in no participant was lesion location isolated to the superior temporal lobe; in all participants the lesion extended into the inferior parietal lobe/temporoparietal junction, eight of the participants has significant middle temporal lobe extension and four of the (most severely affected) participants had further extension into the inferior frontal lobe (Table 1 and Fig. 1).

### Functional magnetic resonance imaging tasks

Successful functional imaging in chronic aphasic participants requires the selection of tasks which are achievable given their level of impairment (Price and Friston, 1999). Accordingly, this study adapted a semantic anterior temporal lobe-activating paradigm used in a previous study with young controls (Visser and Lambon Ralph, 2011) to be suitable for severely impaired participants. The functional MRI tasks consisted of two animate–inanimate judgements tasks using the same items, one in a pictorial and one in a written word modality, and one visual judgement baseline task in which participants judged whether scrambled pictures were high or low on the screen. Each task (picture, word or pattern presentation) was presented in a separate run; they were not mixed because of potential difficulties in task switching that occur in stroke aphasia (Jefferies and Lambon Ralph, 2006). Each run was 9.6 min long and consisted of 16 blocks of stimuli (4 s/stimulus plus 0.5 s prestimulus fixation, total 18 s) interspersed with 16 blocks of rest (18 s). A total of 64 stimuli were presented per run (32 animate, 32 inanimate). Participants responded using a two button response (animate versus inanimate, high versus low). The button box was placed near the participants’ left hand and participants were able to practice responding with this button box before going into the scanner. All pre-scan training was undertaken using a left-handed response. Once a response was made the stimulus was removed from the screen and replaced with a fixation point. The stimuli for the animate-inanimate judgements were the same in both modalities and consisted of eight semantic categories: animate (domestic animals, exotic animals, birds and insects) and inanimate (toys, small household items, clothes and instruments). All stimuli had a spoken word frequency of <20 occurrences per million (Celex database: Baayen *et al.*, 1995) and an imageability of >500 (maximum possible imageability 700: Gilhooly and Logie, 1980; Bird *et al.*, 2001), obtained through the N-Watch database (Davis, 2005). Pictures were black and white and primarily sourced from the Snodgrass and Vanderwart set (1980). Scrambled picture stimuli were created by dividing each stimulus into 80 pieces and randomly redistributing them using the Java Runtime Environment (www.SunMicrosystems.com). Pictures, words and scrambled pictures were centred on either the top or bottom third of the screen. Stimulus presentation was randomized within task and the order of the tasks was randomized between participants. Tasks were delivered using the E-Prime 1.2 software programme (Psychological Software Tools). All control participants received the same training for the three tasks, a total of ∼15 min. The Wernicke’s aphasia participants received individualized packages of training based on their level of impairment. Training was stopped when the Wernicke’s aphasia participants could achieve above 80% accuracy outside the scanner environment on all tasks without external facilitation by the trainer. Stimuli used in the training did not overlap with the experiment stimuli. Inside and outside the scanner, instructions were presented in a pictorial format because of significantly impaired comprehension in the Wernicke’s aphasia group. MR-compatible eyeglasses were provided for those who required them.

### Functional magnetic resonance imaging acquisition

This study used distortion-corrected, spin echo EPI functional MRI. Spin echo sequences produce spatial distortion (but not drop-out) in the anterior temporal lobes and orbitofrontal cortices, which can be corrected using a post-acquisition distortion-correction algorithm (Embleton *et al.*, 2010). The spin echo EPI sequence included 41 slices covering the whole brain with echo time = 70 ms, repetition time = 4150 ms, flip angle = 90°, 96 × 96 matrix, reconstructed resolution 2.5 × 2.5 mm, and slice thickness 3.0 mm. A total of 139 time points were collected each run in a single phase encoding direction (K_L_). To compensate for the distortion, spatial re-mapping correction was applied following spatial realignment; this required the acquisition of an additional scan with the participant at rest, consisting of 20 volumes of interleaved dual direction phase encoding (10 left-to-right phase encoding, K_L_, and 10 right-to-left phase encoding, K_R_). This additional scan was acquired between the first and second experimental run. This method has been successfully applied with non-neurologically impaired participants to reveal semantically-related activations in ventrolateral anterior temporal lobe regions (Binney *et al.*, 2010; Visser *et al.*, 2010*a*) and is described in full elsewhere (Embleton *et al.*, 2010).

### Functional magnetic resonance imaging analysis

#### Preprocessing

Statistical analysis was carried out using the SPM8 software. Preprocessing and general linear model specification were optimized for brains with lesions. The registered, distortion-corrected images were further preprocessed by co-registration to anatomical images and normalization to MNI space using the unified segmentation–normalization procedure (Ashburner and Friston, 2005). This normalization procedure was found to produce optimum results for functional MRI analysis of brains with lesions when medium regularization was applied (Crinion *et al.*, 2007). Following normalization, images were smoothed with an 8 mm full-width at half-maximum Gaussian filter. General linear model analysis is dependent on accurate convolution with the haemodynamic response function. Previous investigations have shown the time-to-peak of the blood oxygen level-dependent response can be significantly delayed in stroke aphasia (Bonakdarpour *et al.*, 2007). The haemodynamic response function time-to-peak was analysed in the Wernicke’s aphasia group using finite impulse response functions and showed no significant deviations in the areas of peak activation. This may be a consequence of peak activation areas being supplied by the non-infarcted posterior cerebral artery (see ‘Results’ section). However, time derivatives were added to the general linear model to account for small deviations in the haemodynamic response function time-to-peak which may be common in elderly participants (D'Esposito *et al.*, 2003) but not observable with a finite impulse response analysis based on a long repetition time of 4150 ms.

### Functional magnetic resonance imaging contrasts

At the first level, whole brain univariate analyses, thresholded at *P < *0.005 with a minimum four-voxel extent, were performed for the semantic animate-inanimate task in each modality. Semantic blocks were contrasted against active baseline blocks and rest blocks. This dual-baseline was considered necessary to ensure that the results were a true reflection of task-related semantic processing. The contrast against baseline blocks accounted for activations related to motor, executive-decision making and visual processes. Rest was used as a contrast in order to account for default processing (such as day-dreaming) which may have been present during the semantic blocks. The task design resulted in periods of rest/fixation during the semantic blocks as semantic items were removed after participant response*.* Default processes engage a network which overlaps with the neural regions of interest to the current study, including anterior temporal regions and further frontal regions of the semantic network. Therefore, this additional rest contrast ensured activations reflected task-related semantic processing.

In the second-level analysis, the task activation coefficient maps described above for pictures and written words were entered into a 2 × 2 mixed-effects ANOVA, with group (Wernicke’s aphasia versus Control) as a between-subject factor and task and the group × task interaction as within-subject factors. Thus, for the main effect of task and the Group × Task interaction, a subject factor was included in SPM’s Flexible Factorial module, whereas this factor was not included for the test of the main effect of Group. Results are displayed for clusters significant at *P < *0.005 uncorrected, minimum cluster size four voxels.

### Region of interest analysis

Further analyses were carried out over *a priori* regions of interest within the sylvian and extra-sylvian semantic network. All regions of interest were bilateral in order to investigate potential reorganization or hemispheric lateralization. Following the procedure described in Visser and Lambon Ralph (2011), regions of interest were derived from independent literatures (see Table 3 for region of interest coordinates and literature sources). Five bilateral region of interest pairs were investigated: (i) anterior fusiform gyri; (ii) temporal poles; (iii) anterior superior temporal gyri/sulci; (iv) ventral occipital-temporal lobe; and (v) inferior frontal gyri. Fusiform gyri, temporal pole and superior temporal gyri regions of interest were selected following previous literature indicating their key involvement in semantic processing in (recovered) aphasia. Inferior frontal gyri regions of interest were selected because of the functional integration between inferior frontal gyri regions and posterior temporal semantic regions damaged in the Wernicke’s aphasia group. The ventral occipital-temporal lobe regions of interest were included because of the consistent neural responses observed in controls in this portion of the ventral visual stream during word recognition and visual object processing (Chao *et al.*, 1999; Twomey *et al.*, 2011). Mean beta weights over the regions of interest were extracted using the MarsBar toolbox (Brett *et al.*, 2002) and further analysed in SPSS. For each region of interest, 2 × 2 ANOVAs were used to investigate main effects of group and condition and group × condition interactions and one-sample *t*-tests were employed to identify regions of significant activation.

**Table 3:** Region of interest analysis coordinates

Region of interest	MNI coordinates			Radius* mm	Coordinate source
	*x*	*y*	*z*		
Left anterior fusiform gyrus	−38	−18	−32	5	Sharp *et al.* (2004)
Right anterior fusiform gyrus	38	−18	−32	5	Homologue
Left temporal pole	−42	16	−32	5	Sharp *et al.* (2004)
Right temporal pole	40	20	−34	5	Sharp *et al.* (2004)
Left anterior superior temporal sulcus	−54	6	−16	7	Scott *et al.* (2000)
Right anterior superior temporal sulcus	54	6	−16	7	Homologue
Left inferior frontal gyrus	−51	30	6	7	Visser and Lambon Ralph (2011)
Right inferior frontal gyrus	50	30	6	7	Homologue
Left ventral occipital-temporal lobe	−38	−44	−18	7	Visser and Lambon Ralph (2011)
Right ventral occipital-temporal lobe	42	−44	−18	7	Visser and Lambon Ralph (2011)

*After Visser and Lambon Ralph (2011) regions of interest were selected from Sharp *et al.* (2004) and Scott *et al.* (2000); the same radii dimensions were used for these regions of interest as in Visser and Lambon Ralph (2011). The larger radius of 7 mm was used for the regions of interest selected from the Visser and Lambon Ralph (2011) results.

## Results

### Behavioural task

The Wernicke’s aphasia comprehension-impaired group was significantly less accurate than the control group on the semantic and baseline tasks. Table 4 displays d’ accuracy scores and reaction time data for the Wernicke’s aphasia and control groups. For the picture task, 10 of 12 participants with Wernicke’s aphasia performed above chance (DL, LS, LB, CB, RD, MC, EL, NM, CH and CW: binomial *P < *0.001 for all participants). For the word condition, eight of the participants with Wernicke’s aphasia performed above chance (DL, CB, RD, MV, EL NM, CH, CW: *P < *0.005). For the control condition, eight of the Wernicke’s aphasia participants performed significantly above chance (DR, DL, RD, MC, EL, NM, CH, CWL: *P < *0.005).

**Table 4:** Functional MRI behavioural task analysis

		Pictures		Words		Scrambled pictures	
		d' (SD)	RT (SD)	d' (SD)	RT (SD)	d' (SD)	RT (SD)
		Max 13.9		Max 13.9		Max 13.9	
	Wernicke’s aphasia	4.9 (5.5)	1460 (422)	4.3 (5.6)	1583 (456)	6.5 (6.0)	1331 (568)
	Control	9.8 (4.8)	929 (187)	11.0 (5.3)	1123 (422)	11.7 (4.2)	749 (205)
							
*t*-test	*t*(22)	2.3	4	3	2.6	2.5	3.4
	*P*	0.03	0.001	0.006	0.018	0.02	0.003

Table displays means and standard deviations for d’ scores and reaction times for each functional MRI tasks, along with independent samples *t*-tests displaying group differences.

RT = reaction time.

### Functional magnetic resonance imaging

Three versions of the functional MRI analysis were run based on the behavioural results: (i) all responses from all participants; (ii) all responses from participants performing above chance; and (iii) only correct responses from all participants. Results from analysis (i) are presented as all analyses yielded similar results. One significant difference occurred in the region of interest analysis between analysis (i) and analysis (ii), reported below.

#### Whole brain analysis results

**Figure 2:**
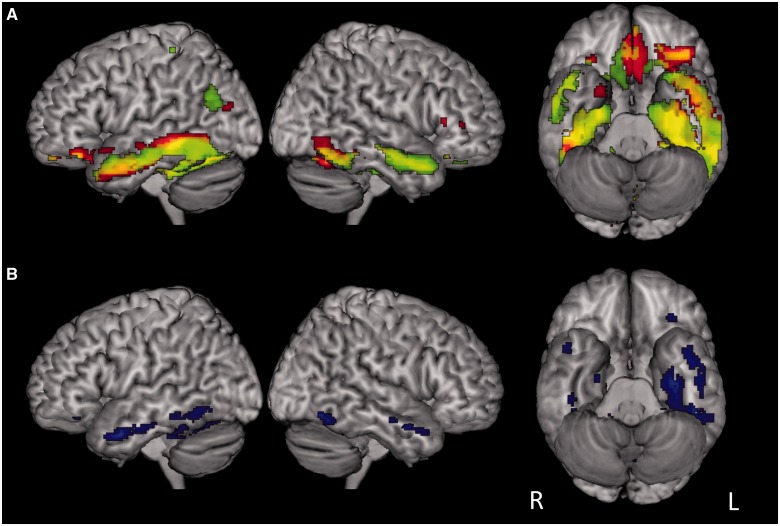
Whole-brain results for semantic condition versus dual baseline. (**A**) significant activation in the Wernicke’s aphasia group for picture (red) and word (green) semantic judgement, overlapping regions in yellow. (**B**) Main effect of group. Regions significantly more active in Wernicke’s aphasia than control group are shown in blue. No temporal lobe regions were more active in the control than Wernicke’s aphasia group. Displayed activations significant at *P < *0.005, uncorrected.

Figure 2 displays whole brain results for the Wernicke’s aphasia group and regions significantly more active in the Wernicke’s aphasia than control group. Peaks for the within-group simple main effects and main effect of group are presented in Tables 5–7.

**Table 5:** Peak whole-brain coordinates for participants with Wernicke’s aphasia

Task	Region	Subregion	Brodmann area	MNI coordinates			*Z*-score
				*x*	*y*	*z*	
Pictures > dual-baseline	Temporal lobe	L. post. middle temporal gyrus	19	−34	−80	20	3.12
		L. ant. middle temporal gyrus	21	−56	−10	−10	2.76
		R. ant. middle temporal gyrus	21	46	0	−22	4.02
		R. temporal pole	38	48	12	−26	3.86
	Frontal lobe	L. medial frontal gyrus	11	−2	26	−14	3.67
		R. medial frontal gyrus	11	2	36	−16	3.84
		R. inferior frontal gyrus	45	50	28	8	3.13
			46	42	40	6	2.96
	Occipital lobe	R. lingual gyrus	17	10	−90	0	3.02
	Cerebellum	L. post. lobe		−34	−64	−18	4.96
		R. post. lobe		8	−52	−6	2.83
Words > dual-baseline	Temporal lobe	L. mid. middle temporal gyrus	21	−60	−42	−12	5.23
		L. post. middle temporal gyrus	39	−40	−66	26	3.71
	Frontal lobe	L. inferior frontal gyrus	47	−34	34	−18	3.78
		L. superior frontal gyrus	11	−20	42	−12	2.93
		L. medial frontal gyrus	11	−2	36	−16	3.06
		R. medial frontal gyrus	11	8	34	−22	3.3
		R. inferior frontal gyrus	47	22	16	−24	2.96
	Parietal lobe	L. angular gyrus	39	−50	−68	34	2.79
	Occipital lobe	L. precuneus	31	−22	−80	26	2.82
		R. lingual gyrus	17	12	−88	−2	3.06
	Cerebellum	L. ant. lobe		−2	54	−20	2.99

**Table 6:** Peak whole-brain coordinates for control participants

Task	Region	Sub-region	Brodmann area	MNI coordinates			*Z*-score
				*x*	*y*	*z*	
Pictures > dual-baseline	Temporal lobe	L. post. fusiform gyrus	19	−38	−74	−18	3.62
	Cerebellum	L. post. lobe		−42	−68	−22	3.03
		R. post. lobe		8	−72	−36	2.98
Words > dual-baseline	Temporal lobe	R. post. fusiform gyrus	19	−38	−74	−18	4.13
		R. mid. fusiform gyrus	37	50	−48	−24	3.28
		L. post. fusiform gyrus	37	−42	−58	−20	3.27
		L. mid parahippocampal gyrus	35	24	−20	−14	2.94
	Frontal lobe	L. middle frontal gyrus	9	−40	12	34	3.88
		R. medial frontal gyrus	25	4	14	−18	3.06
		L. superior frontal gyrus	10	−24	52	30	3

**Table 7:** Peak whole brain coordinates for Wernicke’s aphasia > control

Region	Sub-region	Brodmann area	MNI coordinates			*Z*-score
			*x*	*y*	*z*	
Temporal lobe	L. uncus	20	−32	−14	−26	3.69
	L. ant. middle temporal gyrus	21	−48	2	−28	3.29
	L. ant. inferior temporal gyrus	20	−52	−14	−24	3.28
	L. ant. fusiform gyrus	20	−54	−6	−24	3.05
	R. ant. middle temporal gyrus	21	46	0	−22	3.2
	R. temporal pole	38	48	12	−26	2.99
	R. ant. superior temporal gyrus	21	58	−10	−16	2.72
	R. ant. parahippocampal gyrus	35	24	−10	−22	2.77
	R. mid. fusiform gyrus	20	46	−26	−20	2.71
Frontal lobe	L. middle frontal gyrus	11	−32	36	−16	2.77
Cerebellum	L. post. lobe		−18	−66	−18	3.75
	R. post. lobe		44	−62	−16	3.72

Contrasts of semantic judgements and dual baseline in the Wernicke’s aphasia group produced extensive activation throughout the temporal lobes (Fig. 2). Picture and written word judgements produced activation in the ventral temporal lobes bilaterally. Picture judgements produced activation bilaterally throughout the fusiform extending into the temporal poles; bilaterally in the ventral occipito-temporal region and bilaterally in the anterior superior and middle temporal gyri. Additional peaks were observed in the medial frontal cortex bilaterally. Written word judgements activated the bilateral fusiform gyri, left parahippocampal gyrus; left middle temporal gyrus extending into the temporal pole; right middle and superior temporal gyri and superior temporal sulcus extending into the temporal pole. Further peaks were observed in the left inferior angular gyrus, left inferior frontal gyrus and bilateral medial frontal lobe. In contrast, the control group showed considerably less activation overall with pictures activating the left posterior fusiform gyrus and words producing activation in the posterior fusiform gyri bilaterally and left parahippocampal gyrus. Small additional peaks were observed in the left middle and superior frontal gyri and right medial frontal lobe.

Corresponding to this, the main effect of group was reflected in significantly greater activation in the Wernicke’s aphasia group than the control group throughout the ventral and middle temporal lobes, and to a greater extent on the left than the right. Additionally, the Wernicke’s aphasia group, produced greater activation in the right superior temporal sulcus, extending into the temporal pole. The control group showed significantly greater activation than the Wernicke’s aphasia group only in a single cluster in the right posterior cingulate. Main effects of condition were limited. Pictures produced significantly greater activation in the left ventral temporal pole whereas written words produced significantly greater activation in small clusters in the bilateral anterior parahippocampal gyri and right inferior frontal gyrus. There were no Group × Condition interactions.

#### Region of interest analysis

**Figure 3:**
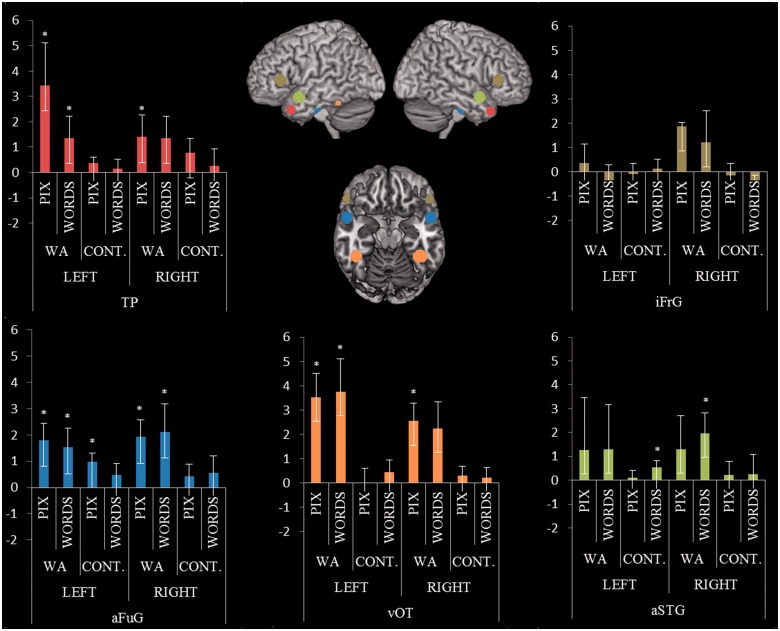
Region of interest analyses. Graphs display mean beta values for each group in each region of interest. Patients with Wernicke’s aphasia (WA) demonstrated significant activation for picture and written word semantic decisions in the anterior fusiform gyrus and ventral occipital-temporal lobe bilaterally and in the left lateral polar region. Written word decision produced additionally significant results in the right anterior superior temporal gyri/sulci. aFuG = anterior fusiform gyrus; aSTG = anterior superior temporal gyrus; TP = temporal pole; vOT = ventral occipital-temporal lobe; iFrG = inferior frontal gyrus. *P < 0.05, one sample *t*-test.

The region of interest analysis (Fig. 3) showed a very similar pattern of results. One tailed one-sample *t*-tests found that control participants significantly activated the left anterior fusiform gyrus for picture semantic judgements [*t*(11) = 3.1, *P = *0.005] and the left anterior superior temporal gyri/sulci for written word judgements [*t*(11) = 1.9, *P = *0.037]. The Wernicke’s aphasia group showed significant activation for both picture and word conditions in the left anterior fusiform gyrus [pictures: *t*(11) = 2.8, *P = *0.009, words: *t*(11) = 2.0, *P = *0.034], the right anterior fusiform gyrus [pictures: *t*(11) = 2.9, *P = *0.008; words: *t*(11) = 1.9, *P = *0.04], left temporal pole [pictures: *t*(11) = 2.0, *P = *0.034; words: *t*(11) = 1.9, 0.04] and the left ventral occipital-temporal lobe [pictures: *t*(11) = 3.6, *P = *0.004; words: *t*(11) = 2.8, *P = *0.017]. The Wernicke’s aphasia group displayed additional activation in the right anterior superior temporal gyrus/sulcus for word stimuli [*t*(11) = 2.27, *P = *0.022] and in the right ventral occipital-temporal lobe for the picture stimuli [*t*(11) = 3.5, *P = *0.005]. Removal of the participants who performed at chance for the picture or written word judgements made no change to significance with the exception that significant activation was additionally found in the right temporal pole for the picture judgements [*t*(9) = 2.1, *P = *0.03]. A ANOVA (2 × 2) revealed main effects of group in the left ventral occipital-temporal lobe [*F*(1,22) = 10.14, *P = *0.004], right ventral occipital-temporal lobe [*F*(1,22) = 5.81, *P = *0.025] and left temporal pole [*F*(1,22) = 4.65, *P = *0.042] caused by significantly greater semantic activation in the Wernicke’s aphasia group than in the control group. There were no significant effects of condition or significant interactions.

## Discussion

By using distortion-corrected functional MRI, we were able to establish that individuals with chronic Wernicke’s aphasia activated extensive areas of the temporal lobe bilaterally while making simple superordinate semantic judgements of written words and pictures. Written words and pictures elicited similar activation profiles, despite greater behavioural impairments in written word comprehension being a characteristic of Wernicke’s aphasia. These results provide further evidence for the crucial role played by the ventrolateral aspects of the anterior temporal lobes in multimodal semantic processing.

### Anterior temporal lobe function in Wernicke’s aphasia

The Wernicke’s aphasia group displayed activation through the inferior temporal lobes bilaterally while making animate-inanimate judgements. Further peaks were identified in additional components of the left hemisphere semantic network in the left inferior frontal gyrus and left posterior temporoparietal junction. The visual ventral stream is thought to be organized as a recurrent hierarchical network that enables the formation of successively complex representations through combining low level visual inputs (Kravitz *et al.*, 2013). Anterior in the ventral stream, the anterior fusiform gyri and temporal poles are highly interconnected to other modality specific processing regions and medial temporal lobe areas (Duffau *et al.*, 2008; Binney *et al.*, 2012). This interconnectivity is thought to allow the abstraction of transmodal semantic representations (Patterson *et al.*, 2007; Lambon Ralph *et al.*, 2010).

The fact that we observed activation in the anterior temporal lobe despite posterior temporal lesions provides further evidence for the key role played by this region in semantic representation in patients and controls (Sharp *et al.*, 2004; Binney *et al.*, 2010; Lambon Ralph *et al.*, 2010; Pobric *et al.*, 2010*a*; Visser and Lambon Ralph, 2011; Visser *et al.*, 2012). Crucially, the Wernicke’s aphasia group showed significantly greater and more extensive activation than the control group throughout the ventral and superior anterior temporal lobe; activation increased in the anterior temporal lobe and other regions following lesions to posterior temporal regions classically associated with semantic representation access. This increased activation was bilateral but to a greater extent on the left. Therefore, the Wernicke’s aphasia group appeared to require greater recruitment of regions associated with both representational semantic and visual representations of concepts to perform the semantic task.

The activation pattern found in the Wernicke’s aphasia group in the current study mirrored that in a previous study of young control participants when performing a more challenging, speeded version of the same semantic judgement task (Visser and Lambon Ralph, 2011). However, the activation in the Wernicke’s aphasia group extended into the temporal poles, particularly in the left hemisphere, which was not observed in either the elderly or young controls. Extension into lateral polar cortex has been observed, however, for more demanding verbal and non-verbal semantic association decisions (Visser *et al.*, 2012).

Given that control participants recruit similar areas under more challenging conditions, the results in the Wernicke’s aphasia group could be interpreted primarily as an enhancement or ‘overactivity’, rather than recruitment of new neural components. Two accounts of enhancement have been proposed: (i) enhancement reflects increased cognitive demands on the region; or (ii) enhancement reflects disinhibition at the neuronal level as a result of deafferentation from another neural region (Price and Friston, 1999). Both of these interpretations can be accommodated in the context of Wernicke’s aphasia and models of semantic processing. Lesions in Wernicke’s aphasia affect both auditory–phonological and semantic processing regions of the posterior language network. In particular, lesions frequently extend into the left angular gyrus, mid and posterior middle temporal gyrus, and underlying white matter (Fig. 1). These regions have been implicated in semantic processing and may support functions such as retrieving and integrating conceptual information (Lau *et al.*, 2008; Binder *et al.*, 2009). Some authors have also suggested that this region is important for extracting conceptual information in relation to events or other time-varying stimuli, thus supporting associative semantic representations (Schwartz *et al.*, 2011). These temporo-parietal junction regions are often found to co-activate with inferior frontal areas during semantic processing (Noonan *et al.*, 2010*,* 2013) and it is proposed that they function within a cognitive control network which, within the semantic domain, may regulate task-directed use of core semantic representations (Jefferies and Lambon Ralph, 2006; Corbett *et al.*, 2009). Therefore, lesions to posterior regions may place greater demands on inferior and anterior aspects of the extra-sylvian temporal semantic network through reduction in semantic processing recourses and/or may lead to disinhibition with these areas through reduced regulation from control processes, leading to overactivation of semantic information irrelevant for task completion. Because the additional activation observed in the inferior temporal lobes in the Wernicke’s aphasia group replicates previous findings from more challenging tasks in control participants, it appears that these are not newly recruited regions. Rather, these extra-sylvian regions are functioning in an inefficient and redundant fashion (Price and Crinion, 2005; Zahn *et al.*, 2006); consequently, maximum processing capacity will be reached more rapidly as task difficulty increases.

### Reorganization of semantic processing

For the written word semantic judgements, the Wernicke’s aphasia group displayed significant activation along the right anterior superior temporal sulcus and superior temporal gyrus (whole brain and region of interest analysis). In comparison, the control participants displayed activation in the homologue region, the left anterior superior temporal gyrus/sulcus, for the same task (region of interest analysis). The left anterior superior temporal sulcus/superior temporal gyrus is associated with the processing of abstract intelligible phonological information in both spoken and written forms (Scott *et al.*, 2006; Spitzyna *et al.*, 2007; Richardson *et al.*, 2011). Dynamic causal modelling of functional MRI data indicates that the left anterior superior temporal sulcus receives input from both higher-order visual regions in the ventral occipital temporal lobe and from auditory-phonological regions in the posterior superior temporal sulcus (Richardson *et al.*, 2011). This converges with psycholinguistic models which propose that written word comprehension (in semi-transparent orthographies such as English) is mediated by a phonological as well as a visual pathway (Plaut *et al.*, 1996). The importance of the right anterior superior temporal gyrus/sulcus in language comprehension subsequent to left posterior temporal lesions has been demonstrated in previous studies of spoken language comprehension and has been related to the degree of recovery from comprehension impairment (Crinion and Price, 2005; Warren *et al.*, 2009). In comparison to previous studies, the current Wernicke’s aphasia group displayed written word comprehension impairments and were significantly less accurate at the functional MRI task than the control group, but still activated the right anterior superior temporal gyrus/sulcus. This may indicate that recovery of function is not only related to which areas of the brain are newly recruited after lesion, but also to the deeper functional organization of those regions with respect to factors including the nature of the stimuli, the precision of perceptual processing of the stimuli and the connectivity of the regions.

It was hypothesized that, because of the additional phonological impairment in Wernicke’s aphasia, the written word semantic judgements might display a greater degree of reorganization than the picture judgements. The results did not support this prediction. The region of interest analysis did not reveal any significant effects of condition and no Group × Condition interaction was observed at the whole brain level. There may be multiple explanations for this. The participants with Wernicke’s aphasia might have relied to a greater extent on the visual reading pathway than the phonological pathway to achieve written word judgements. Conversely, they might have attempted to recruit phonological regions to support the picture semantic judgements, e.g. through sub-vocal naming. Given that written word judgements can be achieved through both visual and phonological routes, further investigation is required to establish whether semantic network recruitment in Wernicke’s aphasia can be dependent on input modality or is multimodal as suggested by the current results.

With the exception of the right anterior superior temporal gyrus/sulcus, the Wernicke’s aphasia group did not recruit any additional regions in comparison with the activation profile observed in young controls undertaking a more demanding version of the same task (Visser and Lambon Ralph, 2011). For example, the Wernicke’s aphasia group did not display significant widespread upregulation of inferior frontal or temporoparietal junction components of the left hemisphere semantic network or their right hemisphere homologues. Overall, there was limited evidence for large-scale reorganization of the semantic network in the chronically impaired individuals who participated in this study. However, given the low semantic demands of the animate-inanimate judgement task, this interpretation must be viewed with caution. Further work investigating more semantically complex judgements is required to investigate organizational changes to the wider semantic system. However, such an approach is highly methodologically challenging due to the significant behavioural impairments associated with more complex processing (see below).

### Methodological considerations

The anterior inferior temporal lobes have often been omitted from traditional neurobiological models of language. This exclusion may have multiple methodological causes (Visser *et al.*, 2010; Lambon Ralph, 2014). The anterior inferior temporal lobes lie outside the territory of the left middle cerebral artery (Phan *et al.*, 2005). As middle cerebral artery territory lesions most frequently lead to aphasia, it is areas within this territory that have been most consistently implicated in language processing, to the exclusion of extra-middle cerebral artery regions. Secondly, functional MRI scanning with gradient echo sequences, the most common neuroimaging methodology, suffers from signal drop-out and distortion in the orbito-frontal cortex and anterior inferior portions of the temporal lobes (Weiskopf *et al.*, 2006). Thirdly, neuroimaging experiments that do not have full brain coverage can cut-off anterior inferior temporal lobe regions, if anterior commissure–posterior commissure oriented acquisition windows/PET cameras are aligned to include the top of the brain (Visser *et al.*, 2010*b*). This study demonstrated semantic processing in the anterior temporal areas with a distortion-corrected spin-echo sequence that compensates for signal dropout in the orbitofrontal cortex and anterior temporal lobes (Embleton *et al.*, 2010).

Task selection is an important consideration when interpreting neuroimaging results wtih neurologicaly impaired populations. It has been emphasized that control and patient groups should be engaging in the same cognitive operations and, therefore, should be undertaking the same task (Price and Friston, 1999). Furthermore, the patient group should be able to perform the task accurately, as a significant degree of error means that the elicited activation cannot be associated reliably with the intended cognitive operations but might also reflect alternative/additional cognitive processes such as error monitoring (Price and Friston, 1999). This study investigated semantic processing of single visually presented items, comprehension of which is relatively spared in Wernicke’s aphasia compared with comprehension of spoken items. However, despite using a task with low executive and semantic demands and extensive pre-scan training, performance in the Wernicke's aphasia group remained worse than that of controls. The Wernicke’s aphasia group was impaired relative to the control group on the baseline as well as the semantic tasks. Although the baseline task has no semantic/linguistic content, this pattern of results is not unexpected and may reflect multiple sources of difficulty. As described above, lesions in Wernicke’s aphasia extend into the posterior components of a frontoparietal executive control network required for efficient task completion, even tasks with limited executive complexity. Additionally, individuals with stroke aphasia have been found to suffer from domain-general attention impairments (Tseng *et al.*, 1993) which may have contributed to the behavioural impairment. Given these task differences between the Wernicke’s aphasia and the control group it is important to note that the additional analyses, where poorly-performing or inaccurate patients responses were removed, generated the same pattern of results.

Finally, although this study addresses individuals with chronic Wernicke’s aphasia, for whom no further rapid behavioural improvement has been observed, it is important to note that five participants with Wernicke’s aphasia were <1 year post-onset at the time of scanning. As such, some limited plasticity-related changes may still occur in these participants and the core findings from this study should be interpreted in this light. To confirm and enhance the interpretation of results from this study, further longitudinal neuroimaging work in aphasia is required.

## Conclusion

This study found significant activation in anterior temporal lobe regions during single-item semantic processing in individuals with classical Wernicke’s aphasia and, to a lesser extent, in elderly control participants. The patients’ reduction in posterior temporal semantic and phonological processing resources increased reliance on extra-sylvian temporal regions. To enhance the interpretation of findings in aphasia neuroimaging, future work should attempt to investigate patterns of response within regions as well as changes to large-scale network recruitment.

## Abbreviation

PALPA: psycholinguistic assessment of language processing in aphasia
